# Lessons learned from nationwide perinatal audit of severe postpartum haemorrhage in the Netherlands

**DOI:** 10.1093/intqhc/mzag119

**Published:** 2026-08-25

**Authors:** Lisa Broeders, Steven V Koenen, Nicole de Vocht, Thomas van den Akker, Ageeth N Rosman

**Affiliations:** Department of Obstetrics and Gynaecology, Leiden University Medical Centre, 2333 ZA Leiden, The Netherlands; Perined, 3528 BL Utrecht, The Netherlands; Department of Obstetrics and Gynaecology, Fam/ETZ, 5000 LC Tilburg, The Netherlands; Perined, 3528 BL Utrecht, The Netherlands; Department of Obstetrics and Gynaecology, Leiden University Medical Centre, 2333 ZA Leiden, The Netherlands; Athena Institute, Faculty of Science, VU University Amsterdam, 1081 HV Amsterdam, The Netherlands; Perined, 3528 BL Utrecht, The Netherlands; Department of Healthcare Studies, Rotterdam University of Applied Sciences, 3011 WN Rotterdam, The Netherlands

**Keywords:** postpartum haemorrhage, perinatal audit, pregnancy complications

## Abstract

**Background:**

Severe postpartum haemorrhage (PPH) remains a leading and largely preventable cause of maternal morbidity and mortality worldwide. Delayed recognition, inappropriate fluid replacement, and suboptimal clinical management contribute to adverse outcomes. Clinical audit can identify deficiencies in care and stimulate quality improvement. In the Netherlands, a nationwide perinatal audit system enables multidisciplinary review of maternity care to enhance collaboration and optimize clinical practice. This study reports the first nationwide audit of severe PPH management in the Netherlands and aims to identify improvable care factors and draw lessons for clinical care.

**Methods:**

In the Netherlands, perinatal audit is nationally coordinated and based on a set of periodic clinical themes. In 2020–3, one such theme was severe PPH, defined as blood loss of ≥2500 ml in the first 24 h after birth or blood loss requiring hysterectomy. Local multidisciplinary audit sessions were held within ‘perinatal cooperation groups’, comprised maternity care professionals working within a region. During audit sessions, anonymized case reports were reviewed, and areas for improvement of clinical care were identified and documented. Audit findings were analysed quantitatively to group improvable care factors regarding clinical management provided to women sustaining severe PPH. In addition, narrative excerpts from audit reports were used descriptively to provide context to the findings.

**Results:**

In 116 women sustaining severe PPH, the audit revealed 234 improvable care factors, mostly related to nonadherence to guidelines and standards (47.8%), organization of care (22.3%), and communication and documentation between care professionals and patients (17.1%). Common issues included absence or ambiguity of local management protocols, unclear allocation of responsibilities among team members, limited capacity in terms of staffing and available labour rooms, and incomplete communication and documentation around referral between/within echelons. Nearly half of the improvable care factors were stated by local audit panellists to be likely or possibly associated with severe PPH.

**Conclusion:**

Audit of PPH care revealed several areas for improvement in the Netherlands, including ensuring sufficient staffing and labour room capacity and updating of national and local management protocols.

## Introduction

Severe postpartum haemorrhage (PPH) remains a significant contributor to maternal mortality on a global scale [1–3]. Therefore, the World Health Organization (WHO) calls for action in its roadmap to reduce deaths from PPH between 2023 and 2030 [4]. Deaths from PPH are generally considered preventable [2, 5]. Delayed recognition, inappropriate fluid replacement, and late or inappropriate clinical management all contribute to PPH-related deaths and disabilities [6]. To achieve better maternal outcomes, access to a well-functioning health system with skilled healthcare providers delivering effective emergency maternity care with timely clinical intervention plays a crucial role in minimizing the impact of PPH.

Previous research demonstrated that regular clinical audit of severe PPH can be associated with a reduction in severe PPH-related outcomes [5, 7]. In the Netherlands, a nationwide system of systematic perinatal audit was introduced in 2010 [8]. As part of this national perinatal audit, maternity care is systematically reviewed and quality of care analysed within local multidisciplinary teams, in order to identify relevant lessons for clinical care. These audit sessions aim to strengthen inter- and transdisciplinary collaboration by jointly reviewing specific case histories and identifying care processes that require improvement [9, 10].

This study reports on the first nationwide audit of severe PPH management and aims to identify areas for improvement with regard to the quality of care provided to women sustaining severe PPH.

## Materials and methods

### Design

This was a quantitative audit study of improvable care factors in the care provided to women sustaining severe PPH in the Netherlands between 2020 and 2023. Narrative information from audit reports was used descriptively to illustrate and contextualize the quantitative findings.

### Setting

In the Netherlands, birth care for low-risk women (primary care) is provided by midwives, whereas in case of medical complications and/or prior increased risk of complications, care is provided at the hospital under supervision of an obstetrician (secondary care) or by clinical midwives under the responsibility of the obstetrician. In case of ante-, intra-, or postpartum complications, women are referred by primary care midwives to the local hospital. A perinatal cooperation group (PCG) comprises primary and secondary healthcare professionals specializing in maternity care. Collectively, they are responsible for monitoring, safeguarding, and enhancing the quality of obstetric care delivered within their PCG. At the time of the study, 71 PCGs were active in the Netherlands, and local audit sessions were conducted within these PCGs.

The nationwide perinatal audit has been coordinated and supported by Perined, with selected clinical conditions audited periodically. Details about the methodology of the perinatal audit in the Netherlands have been published before [11]. Severe PPH was selected as one of the clinical conditions for audit sessions held between 2020 and 2023. Severe PPH was defined as blood loss of ≥2500 ml or requiring hysterectomy (removal of the uterus), as established by the national audit committee.

Before each audit session, local audit teams prepared anonymized (both for women’s and health workers’ characteristics) chronological reports of the severe PPH cases selected for discussion. The local audit team comprised obstetric nurses, community and hospital-based midwives, obstetricians and paediatricians. The chronological report included detailed information about the pregnancy, birth, medical history, and some theme-specific information (e.g. amount of blood loss, most likely cause of blood loss, and interventions performed to control the bleeding). This information was entered into a national database, the Perinatal Audit Application (PAA).

During audit sessions, participants of all relevant cadres (obstetricians, community and hospital-based midwives, paediatricians, obstetric nurses, ambulance staff, pathologists, anaesthesiologists, and maternity care assistants) reviewed an anonymized case history of a woman sustaining severe PPH. The care provided was critically evaluated, and areas for possible improvement were identified. These areas for improvement were then categorized by the audit participants according to the predefined classification outlined in Textbox 1, which was previously developed by van Diem *et al.* [11, 12]. Improvable care factors were classified into three main categories: (i) guidelines, standards, and usual care; (ii) organization of care; and (iii) communication and documentation. The category guidelines, standards, and usual care comprised improvable factors identified in routine clinical practice and based on local protocols. Based on the multidisciplinary discussion, local audit coordinators entered women’s characteristics and the identified ‘improvable care factors’ into the PAA. In addition, audit participants categorized the potential contribution of each improvable care factor to the occurrence of severe PPH as ‘none/unlikely’, ‘possible’, ‘very likely’, ‘unknown’, or ‘no consensus’. This categorization was also entered into the PAA.

Textbox 1 Classification of improvable care factors.

Within perinatal audit, improvable care factors are classified into the following categories:

**Guidelines, standards, and usual care** 

- Deviation from national guidelines
- Deviation from local protocol or adaptation of local protocol
- Deviation from usual care

**Organization of care** 

- Delayed diagnostics, treatment, and/or interventions
- Failing alarm systems
- Inadequate/suboptimal diagnostic processes
- Inadequate/suboptimal supervision
- Organizational problems; unclear coordination and/or responsibilities, possibly caused by staff shortages, skill mismatches, and inefficient workflows

**Communication and documentation** 

- Documentation errors or incomplete entries related to baseline information (e.g. counselling, instructions, contact, and appointments), diagnostic processes, policies and decision-making, and the transfer of care.
- Inadequate information sharing and communication regarding the transfer of care or consultation between/within echelons and the internal transfer of care or consultation within the same profession.
- Women not adequately informed due to poor communication and missing information.

### Inclusion

Women were eligible for audit when their gestational age was at least 24 weeks and met the criteria for severe PPH. Severe PPH was defined as blood loss of ≥2500 ml or requiring a hysterectomy.

### Data analysis

All documented characteristics and improvable care factors were collected and archived in the PAA at Perined. Data were checked for missing values, and where possible, information was added based on the provided chronological reports. Women’s characteristics and improvable factors were summarized using descriptive statistics. Analysis was primarily quantitative. Qualitative data were not subjected to qualitative coding or thematic analysis and were used only for descriptive illustration.

In addition, data on the national population of women who experienced PPH with a blood loss of ≥2.5 l in 2023 were obtained from a separate national database maintained by Perined. These data were used to describe and compare characteristics of the study population with those of the national population with severe PPH, using standardized mean differences (SMDs) and absolute differences. SMD were interpreted as <0.10 (negligible), 0.10–0.20 (small), and >0.20 (meaningful imbalance).

## Results

### Characteristics

In total, 116 case histories of severe PPH were audited. Of these 116 women, 14 (12.1%) were classified as obese (body mass index ≥30), 10 (8.6%) had given birth before the 36th week of gestation, and 6 (5.2%) were 40 years or older. A total of 64 women (55.2%) were multiparous, of whom 27 (41.2%) had sustained blood loss of more than 1 l in their obstetric histories (Table 1). In our study, 32 women (27.6%) had had an induction of labour and 28 (24.1%) underwent labour augmentation. Most women gave birth vaginally (76.7%). The second stage of labour in 26 women (25.0%) took 60 minutes or longer.

**Table 1:** Baseline characteristics of women with severe postpartum haemorrhage (≥2500-ml blood loss) and/or hysterectomy.

		Study population		National population of women with PPH ≥2.5 l in 2023		Absolute difference (%)	SMD
		*n*	%	*n*	%		
Total		116		1197			
Age (years)	<30	30	25.9	338	28.2	2.3	0.171
	30–34	50	43.1	520	43.4	0.3	
	35–39	30	25.9	269	22.5	3.4	
	≥40	6	5.2	57	4.8	0.4	
	Missing	0	0.0	13	1.1		
BMI^a^	<25	76	65.5	468	39.1	26.4	0.911
	25–29.9	26	22.4	253	21.1	1.3	
	≥30	14	12.1	145	12.1	0.0	
	Missing	0	0.0	331	27.7		
Ethnicity	White	102	87.9	974	81.4	6.5	0.218
	Non-White	13	11.2	183	15.3	4.1	
	Missing	1	0.9	40	3.3	2.4	
Parity	Nulliparous	52	44.8	573	47.9	3.1	0.262
	Multiparous	64	55.2	589	49.2	6.0	
		0	0.0	35	2.9		
Gestational age	24w0d–36w6d	10	8.6	131	11.5	2.9	0.078
	≥37w0d	106	91.4	1,066	89.1	2.3	
Caesarean section in history^b^		3	4.7	70	11.9		
Manual removal of placenta in history^b^		2	3.1	30	5.1		
PPH^c^ in history^b^		26	40.6	19	3.2	37.4	1.013
Augmentation		28	24.1	553	51.3	27.2	0.585
Induction of labour		32	27.6	427	35.7	8.1	0.175
Mode of birth	Vaginal birth	81	69.8	917	76.6	6.8	0.234
	Assisted vaginal birth	8	6.9	97	8.1	1.2	
	Emergency caesarean section	15	12.9	84	7.0	5.9	
	Planned caesarean section	12	10.3	96	8.0	2.3	
Duration of second stage of labour^d^	<30 min	48	41.4	508	42.4	1.0	0.055
	30–60 min	22	19.0	202	16.9	2.1	
	≥60 min	26	22.4	275	23.0	0.6	
	Unknown	20	17.2	212	17.7	0.5	

a Body mass index (kg/m^2^).

b Only multipara.

c Postpartum haemorrhage (blood loss >1000 ml).

d Planned caesarean section excluded.

Baseline characteristics of the audited cases were compared to those of the national population of women with postpartum blood loss ≥2.5 l (*n* = 1197). Negligible to small differences were observed for age (SMD = 0.171), gestational age ≥37 weeks (SMD = 0.078), and induction of labour (SMD = 0.175). Meaningful imbalances (SMD >0.20) were found for ethnicity (SMD = 0.218), parity (SMD = 0.262), history of caesarean section (SMD = 0.263), mode of delivery (SMD = 0.234), body mass index (BMI) (SMD = 0.911), labour augmentation (SMD = 0.585), and previous PPH (SMD = 1.013). Compared with the national population of women with PPH ≥2.5 l in 2023, the audited cases had higher proportions of PPH in history and a lower proportion of augmentation.

### Improvable care factors

A total of 234 improvable care factors were formulated and divided into subcategories (Table 2). Most improvable care factors were related to nonadherence to guidelines or other clinical standards (112; 47.9%). Additionally, 52 improvable care factors (22.2%) fell into the category ‘organization of care’ and 40 (17.1%) factors were related to communication; 30 (12.8%) of the improvable care factors were categorized as ‘other’.

**Table 2:** Improvable care factors.

	Total (*n* = 234)	
Improvable factors per case^a^	**2 (1–8)**	
Type of improvable factor		
**Guidelines and standards**	**112**	**47.9%**
Deviation from standard care	22	9.4%
Deviation from national guidelines	20	8.5%
Deviation from local guidelines	41	17.5%
Other areas for improvement in guidelines and standards	29	12.4%
**Organization of care**	**52**	**22.2%**
Delay	16	6.8%
Failing alarm systems	1	0.4%
Inadequate diagnostics	7	3.0%
Inadequate supervision	2	0.9%
Organizational problem	24	10.3%
Technical problem	2	0.9%
**Communication and documentation**	**40**	**17.1%**
Communication problem	26	11.1%
Inadequate documentation	14	6.0%
**Other**	30	12.8%
Other areas for improvement	30	12.8%

a Multiple improvable care factors may apply to a single audit case.

### Guidelines, standards, and usual care

Improvable care factors related to guidelines, standards, and usual care were identified in a substantial proportion of cases and, where possible, clustered into two categories: protocol absence or ambiguities and nonadherence. Ambiguities in protocols included absence of recommendations for nursing actions related to PPH, unclear escalation thresholds, and undefined responsibilities. Nonadherence to the protocol included omitting recommended interventions, such as administering uterotonics or bladder catheterization. Nonadherence can result from the acute setting, patient preference, insufficient health worker knowledge, or because the protocol is unclear (see Textbox 2 for illustrative quotes).

Textbox 2 Improvable care factors related to guidelines and standards.

**Protocol absence and ambiguity** 

*‘No clear guideline exists for the sequence of actions in managing PPH in community care settings’.*

*‘The procedure for receiving blood products during severe PPH is perceived as overly complex, placing excessive demands on staff who must simultaneously deliver acute patient care’.*

*‘There is uncertainty about when someone should or should not be referred for consultation to secondary care, when there is a PPH of less than 2000 ml, because the protocol is not entirely clear’.*

**Nonadherence to protocols** 

*‘The regional protocol was not properly followed: no uterine massage was performed, the bladder wasn’t emptied, no intravenous fluids were administered, an additional 5 units of oxytocin were not given, oxygen was not given, and parallel actions were not initiated’.*

### Organization of care

Improvable care factors in the category organization of care concerned internal procedures, workflow, and team coordination. These included inadequate or unclear organizational structures and processes, leading to confusion and inefficiencies that compromised the continuity and quality of patient care. For example, inadequate preparation of the patient for transfer, late or incomplete notification of anaesthesiology staff, and ambiguous allocation of responsibilities between team members. Logistical challenges were also mentioned, including limited staffing and labour rooms, unfamiliarity of ambulance personnel with hospital routing and incorrect use of emergency alarm systems (see Textbox 3 for illustrative quotes).

Textbox 3 Improvable care factors related to organization of care.

**Unclear responsibilities and coordination** 

*‘The woman was in the recovery room after surgery until she was stable enough to return to the ward. However, the nurse in the recovery room did not look under the covers to monitor blood loss. When the nurse from the department came to take her, she was lying in a pool of blood and nothing had been done to stop the bleeding and consult the obstetrician that treated her’.*

**Limited capacity** 

*‘Busyness in the operating room, resulting in no operating room or staff being available. There was a delay of 1 hour after registering for manual placental removal before access to the operating room was available’.*

*‘Referral was declined by another hospital due to capacity constraints, despite a critical indication of severe postpartum haemorrhage (PPH > 2 liters)’.*

### Communication and documentation

Inadequate or incomplete communication and documentation were identified during the transfer of care between professionals, resulting in missing information and compromising the continuity and quality of care. Improvable care factors in this category involved both communication and documentation during the transfer of care within one facility (either a hospital department or a specific midwifery practice) as well as between professionals from different facilities.

Challenges were particularly evident during emergency situations, where information exchange was often fragmented and documentation incomplete. Handover between care levels was frequently unclear or insufficiently structured. In several cases, patients were transferred from other locations without comprehensive antenatal information or a clear clinical summary. Relevant clinical questions and key information were not always explicitly communicated. These shortcomings may compromise continuity of care, cause delays in important decisions, and increase the chance of mistakes (see Textbox 4 for an illustrative quote).

Textbox 4 Communication and documentation.

**Communication and documentation during the transfer of care** 

*‘It was not clear to everyone how much the total blood loss was at what time in a woman with severe PPH that progressed slowly and involved multiple healthcare providers (and therefore multiple moments of transfer of care)’.*

*‘The conclusions from the clinical policy discussion were not documented in the patient medical record’.*

### Other areas for improvement

A total of 30 improvable care factors identified during perinatal audit could not be categorized in any of the above categories. These included, for example, reminding healthcare professionals of the possibility of contacting the Guidance and Support Team when additional support was required. In one instance, a pregnant woman’s extensive birth plan was not properly transferred during transfer of care and was therefore not incorporated into the clinical management plan. In another case, therapeutic anticoagulation was prescribed shortly before delivery in a pregnant woman. Provision of timely postpartum follow-up care for women after a complicated birth to prevent psychological trauma was also identified as an improvable factor.

### Improvable care factors and probable association with outcome

Of all improvable care factors identified during the nationwide perinatal audit, 114 (48,7%) were identified as (very) likely or possibly related to the outcome of severe PPH (Table 3). The majority of these factors were related to guidelines and standards.

**Table 3:** Improvable care factors and probable relation with outcome.

Probable relation with outcome	Total (*n* = 234)	
(very) Likely	31	13.2%
Possible	83	35.5%
None/unlikely	106	45.3%
Not assessable	10	4.3%
No consensus	4	1.7%

## Discussion

### Statement of principal findings

This nationwide perinatal audit on severe PPH provides insight into improvable care factors that could improve birth care in the Netherlands. It highlights opportunities for improvement in communication, treatment delays due to organizational problems, and the implementation of and adherence to appropriate clinical guidance.

### Interpretation within the context of the wider literature

Similar improvable care factors were found previously in the Netherlands. Woiski *et al.* acknowledged ambiguity and incompleteness in PPH protocols and problems within the domain of communication [13]. Ramler *et al.* studied all obstetric haemorrhage-related deaths between 2006 and 2019 and highlighted the need for more structured and effective communication, early recognition of persistent bleeding, prompt involvement of a senior clinician, and timely, tailored management of haemorrhage with attention to coagulopathy [14]. A similar audit on maternal deaths was carried out in Italy, identifying comparable areas for maternity care improvement [5]. Moreover, previous articles discussing lessons learned from the Dutch perinatal audit of other clinical conditions [15–17] have consistently pointed out communication and organizational problems. Consistent and effective follow-up on improvable care factors identified during audit is crucial to achieve sustainable improvements in maternity care. Greater support and focus should be given to translate the identification of improvable care factors into actual action.

### Strengths and limitations

A limitation of this study is the use of a blood loss threshold of 2.5 l to define severe PPH. Although this threshold was predefined within the national audit framework, blood loss estimation is known to be imprecise and may be subject to substantial measurement error. It should also be noted that visual estimation frequently underestimates actual blood loss, particularly in cases of severe haemorrhage. Consequently, some women with clinically significant PPH may not have met the inclusion criterion, while others may have been misclassified with respect to severity. Alternative definitions based on more objective indicators, such as the requirement for a predefined number of packed red blood cell transfusions, may provide a more robust measure of severe PPH. Selection bias cannot be excluded, as cases included in the audit were not randomly selected from all eligible women. To assess the representativeness of the audited cohort, we compared its characteristics with those of the national population of women with postpartum blood loss ≥2.5 l (hereafter, the national PPH population). This comparison has several limitations: peripartum hysterectomy is not recorded in the Perined registry, the comparison period differed from the audit period because of data availability, and several relevant medical history variables are incompletely registered in Perined. Therefore, observed differences between the audited and national PPH population may be influenced by both incomplete registration in the national PPH population and the selective nature of the audit cohort. Nevertheless, several characteristics showed only negligible or small differences between the audit and national PPH populations. Meaningful differences were observed for several characteristics, particularly for labour augmentation and previous PPH. The large difference observed for BMI should be interpreted with caution, given the substantial difference in completeness of BMI registration between the audit and national PPH populations. Although these findings suggest that the audit population may not be fully representative of the national population of women with postpartum blood loss ≥2.5 l, representativeness was not the primary aim of the audit. The purpose was to evaluate and improve the quality of care in severe PPH cases, and the identified areas for improvement are likely to remain relevant despite differences between the audited and national PPH population.

In the Netherlands, PCGs are recommended to audit the records of a maximum of two women for the national perinatal audit. Consequently, not all women who experienced a severe PPH were audited. Selection bias is therefore inherent to the design of this study, as cases might have been chosen based on the potential impact on women and healthcare providers, combined with a desire to implement local policies to prevent similar occurrences. The purpose of the national perinatal audit is just that: to raise awareness of areas for possible improvement of maternity care. It does not aim to be exhaustive in covering all severe PPH happening in the country.

A limitation of this study is the fact that the improvable care factors provided through audit were frequently insufficiently detailed in the PAA. For example, it was regularly unclear whether deviations reflected missing or incomplete protocols, or whether care providers were unable to adhere to existing protocols for practical, knowledge-based, or situational reasons, highlighting the difficulty of classifying some improvable care factors. Improvable care factors should clearly outline the events, context, and roles involved. It is crucial for all audit participants to collectively share the responsibility of identifying and appropriately articulating improvable care factors. Incorrect categorization distorts the outcomes and diminishes the quality of the national database. Furthermore, all cases were reviewed by local audit teams without subsequent centralized reassessment by an independent expert committee. Although audits were conducted according to a national framework with predefined classifications, differences in interpretation between local audit teams may have influenced the identification and categorization of improvable care factors, potentially contributing to variability in the findings. More recently, initial efforts have been undertaken to train teams in formulating clear improvement objectives according to the SMART (Specific, Measurable, Achievable, Relevant, and Time-bound) criteria and in developing and executing implementation plans using the ACTion methodology [18].

### Implications for policy, practice, and research

This study highlights the need for clearer and more consistent national and local PPH protocols, alongside strengthened implementation in clinical practice. Efforts should focus on enhanced team coordination and structured communication. Evidence has highlighted persistent challenges related to communication and organization [5, 13–17]. Consistent and effective follow-up of actions related to identified improvable care factors is essential to achieve sustainable improvement. Greater emphasis is needed on translating audit findings into concrete actions. Future research should explore barriers to guideline adherence and effective communication in PPH care.

## Conclusion

The fact that areas for care improvement have remained similar in the Netherlands over the past decades highlights the central challenge to translate audit findings into effective and sustainable improvement of clinical practice. We hope that, this time around, findings will contribute to lasting and sustainable improvements in maternity care in the Netherlands and elsewhere.

## Data Availability

The data that support the findings of this study are available from Perined (contact via info@perined.nl) for researchers who meet the criteria for access to these confidential data, and only upon Perined’s permission. Secondary data users must sign a formal legal and ethical agreement, respecting all privacy regulations for all study participants.
